# Atypical Hepatocellular Neoplasm With Peliosis in Cirrhotic Liver Versus Hepatocellular Carcinoma: A Diagnostic Trap

**DOI:** 10.1097/MD.0000000000001189

**Published:** 2015-07-24

**Authors:** Simona Gurzu, Ioan Jung, Anca Otilia Contac, Mihai Turcu, Adrian Tudor

**Affiliations:** From Department of Pathology, University of Medicine and Pharmacy, Tirgu-Mures, Romania (SG, IJ, AOC, MT); and Department of Surgery, University of Medicine and Pharmacy, Tirgu-Mures, Romania (AT)

## Abstract

Atypical hepatocellular neoplasm (AHN) is an adenoma-like hepatic tumor that even occurs in noncirrhotic liver of males (any age) or females ≥50 years old, or associates focal atypical features.

In this article, 2 unusual cases diagnosed in elderly cirrhotic patients, unrelated to steroids, are presented.

The first case was incidentally diagnosed in an 83-year-old female. During laparoscopic surgery for cholecystectomy, hemoperitoneum was installed and laparotomy was necessary to remove a 70-mm nodular encapsulated hepatic tumor that was microscopically composed by hepatocyte-like cells with clear cytoplasm, arranged in 1- to 2-cell-thick plates and intermingled with areas of peliosis, negative for alpha fetoprotein (αFP), p53, and keratin 7, with low Ki67 index and intact reticulin framework. The second case was incidentally diagnosed at ultrasound examination in a 66-year-old male. The surgical specimen was a 50-mm solid multinodular tumor that microscopically consisted of 3-cell-thick plates of hepatocyte-like cells with acinar, pseudoglandular, and trabecular architecture, intermingled with peliotic areas, without nuclear atypia and disintegrated reticulin framework. Both of the cases occurred in cirrhotic liver. The tumor cells were marked by AE1/AE3 keratin, displayed a Ki67 index < 5% and were negative for αFP, p53, and keratin 7. No recurrences or any other disorder occurred 6 months after surgery.

In cirrhotic liver, adenomas with peliosis that do not satisfy all the diagnosis criteria synthesized in the article should be considered AHNs and differential diagnosis includes hepatocellular carcinoma but also focal nodular hyperplasia, regenerative nodules, and dysplastic nodules. This histological entity is not yet included in the WHO Classification list.

## INTRODUCTION

Hepatic or hepatocellular adenoma (HA), also known as benign hepatoma, is a rare benign tumor that represents <3% of all primary tumors of the liver^1^ and occurs in 95% of the cases in women younger than 45 years taking oral contraceptives; the annual incidence is 3 to 4 cases/100,000 females.^2–6^ The first case of HA was described by Franco et al in 1952 as an encapsulated liver tumor without bile ducts,^7^ whereas, in 1973, Baum et al evolved the hypothesis about possible association between HA and contraceptive pills consumption.^8^ Later, it was proved that cessation of consumption of steroid hormone-based pills could lead to spontaneous tumor regression.^9^

HAs are considered tumors of noncirrhotic liver that are unusual in males (any age) and women <50 and >15 years old, and their differential diagnosis is very difficult.^2,3,10,11^ Except the patient's age and gender, HAs in the setting of cirrhosis can be diagnosed in only those cases that really satisfy all the diagnostic criteria very stringently (Table 1). Moreover, in adults (males at any age and females >50 years) and also in cases with focal atypical features (Table 1) the diagnosis of atypical hepatocellular neoplasm (AHN) is recommended.^11^

**TABLE 1:**
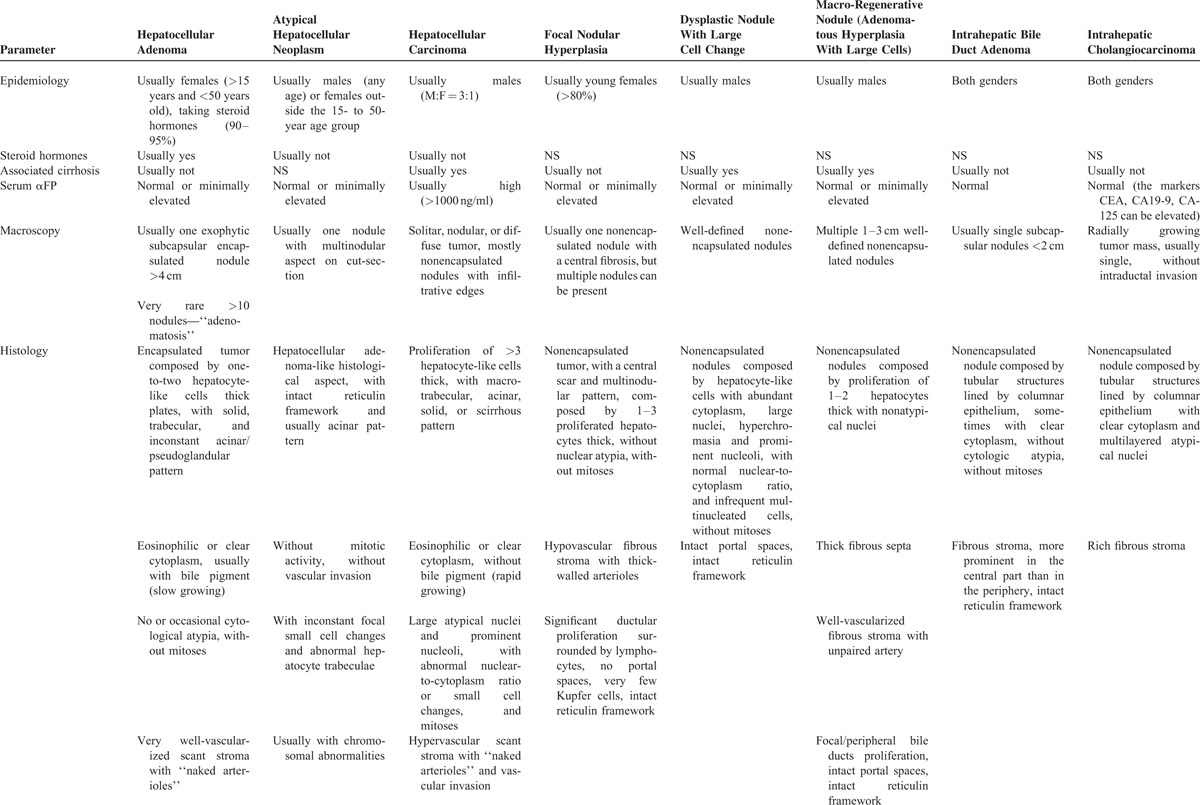
Differential Diagnosis Between Hepatic Adenomas and Other Primary Lesions in Cirrhotic Liver^1–16^

**TABLE 1:**
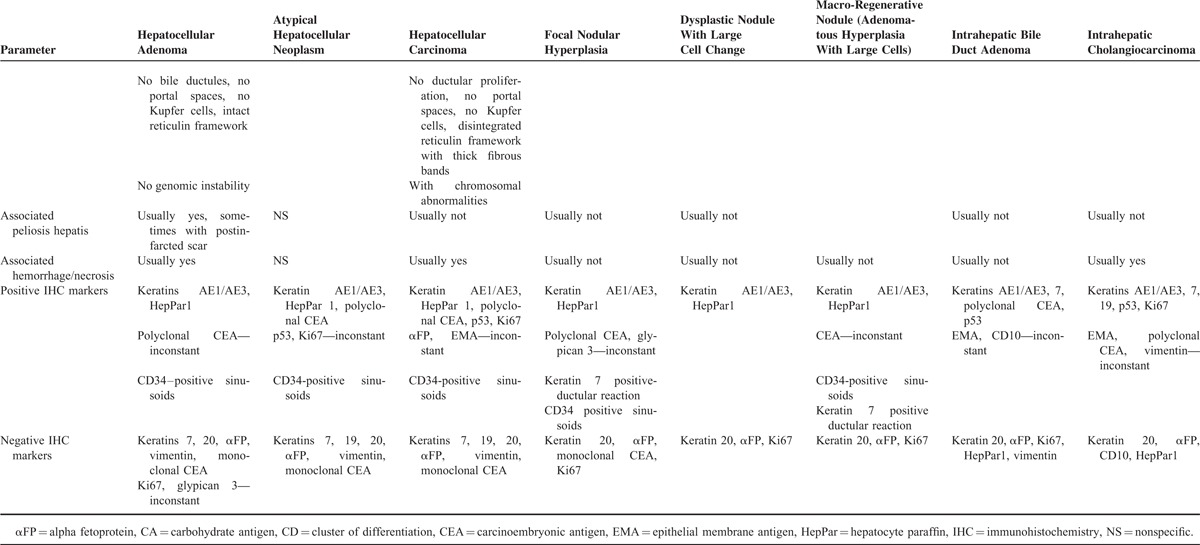
Differential Diagnosis Between Hepatic Adenomas and Other Primary Lesions in Cirrhotic Liver^1–16^

In this article, we present 2 cases that were first diagnosed as HAs with associated peliosis, developed in the hepatitis B-related cirrhotic liver of 2 elderly patients, with high risk of bleeding. Further careful examination leaded to modified diagnosis: one of the cases was classified as AHNs and the other one was considered well-differentiated hepatocellular carcinoma (HCC). Besides the clinicopathologic characteristics of the cases, the criteria of differential diagnosis of HA versus AHN and HCC were presented with illustrative pictures. Consent of the patients was obtained for surgical intervention and publication of these cases.

## PRESENTATION OF CASES

### Case 1

An 83-year-old nonalcoholic female, with 6 months history of lithiasic cholecystitis, was admitted to the hospital with severe right upper quadrant and epigastric pain, nausea, vomiting, and scleral jaundice. Emergent laparoscopic cholecystectomy was decided. Before surgical intervention, ultrasound examination was performed and a hepatic hemangioma of the V, VI, and VII segments of the liver was suspected.

Her past medical history included nonoperated acute pancreatitis (10 years before) and hepatitis B-related Laennec's atrophic cirrhosis (diagnosed 6 years before; at the moment, in compensated status). Five years ago, she was also diagnosed with a right breast neuroendocrine invasive tumor in pT2N0 stage; right mastectomy was performed, without any postoperative oncologic therapy. No recurrences or metastases were suspected. The gynecologic examination revealed no modifications.

At the current admission, blood tests did not show significant disorders except slight anemia (hemoglobin 10.40 g/dL and hematocrit 29.90%), lymphopenia (18.50%), and slight elevated aspartate aminotransferase and alanine transaminase (AST 52.00 U/L, ALT 83.00 U/L).

During laparoscopic surgery, exploration of the peritoneal cavity revealed hemoperitoneum. Laparotomy was performed and the distended gallbladder was removed. The peritoneal hemorrhage occurred as a result of rupture of a 75-mm diameter nodular tumor that was relatively well defined and involved the segments 5, 6, and 7 of the liver.

**FIGURE 1:**
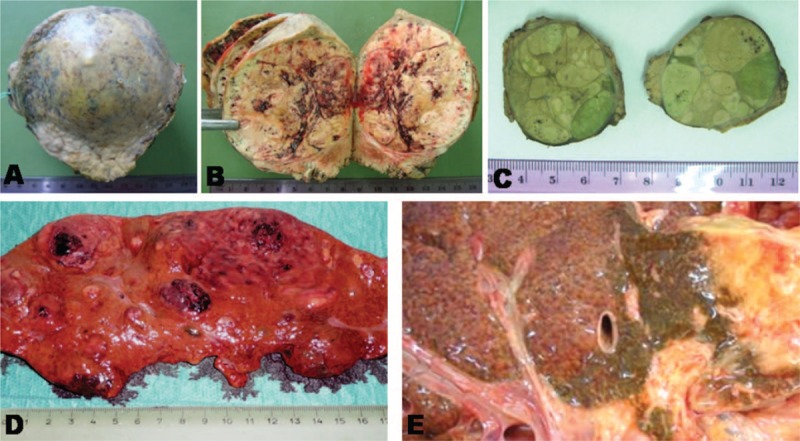
Gross findings of hepatic tumors from authors’ collection. (A and B) Atypical hepatocellular neoplasm—case 1; (C) encapsulated hepatocellular carcinoma—case 2; (D) multifocal hepatocellular carcinoma; (E) cholangiocarcinoma.

**FIGURE 2:**
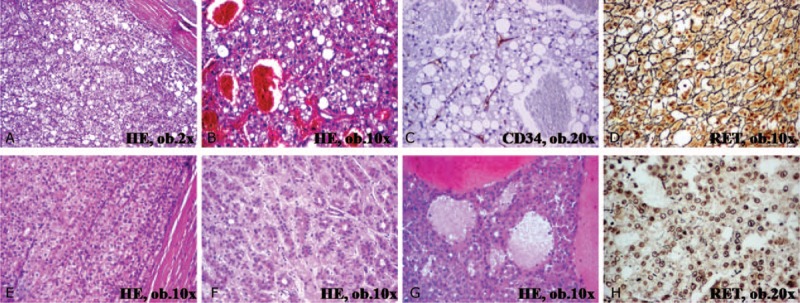
Microscopic features of the hepatic tumors of the cirrhotic liver. In case 1 (A–D), an atypical hepatocellular neoplasm diagnosed in an 83-year-old female, the tumor was covered by a connective capsule (A) and the proliferating cells with clear cytoplasm (A) were intermingled with large blood-filled spaces surrounded by hepatocytes, without lining endothelial cells (B and C); the reticulin (RET) framework is intact (D). In case 2 (E–H), an well-differentiated hepatocellular carcinoma diagnosed in a 66-year-old male, the proliferating cells show trabecular (E) and acinar architecture with clear cytoplasm (F), with large peliotic areas (G), and disintegrated RET framework (H).

Gross examination of the surgical specimen showed a 75 mm × 70 mm × 55 mm encapsulated nodular tumor, with a 25 × 20 ruptured bleeding area. On cut section, the gray-colored tumor had a soft consistency and presented multiple hemorrhagic areas (Figure 1). The liver parenchyma adjacent to the tumor capsule had a nodular aspect and hard consistency. Histopathological examination revealed that the tumor was composed by closely packed hepatocytes delineated by the liver parenchyma through a connective capsule. The tumor cells, which displayed a solid architecture, were arranged in one-to-two closely packed rows, presented irregular cell boundaries, and a rich cytoplasm. Among the hepatocytes, several CD34-positive sinusoidal spaces intermingled with randomly distributed CD34-negative large blood-filled spaces and cysts surrounded by hepatocytes, suggestive for intratumor peliosis, were noted (Figure 2). At high power view, predominately clear cell cytoplasm was noted. Nuclear-to-cytoplasmic ratio was normal, no mitotic figures or nuclear polymorphism was seen. The tumor cells were marked by AE1/AE3 keratin and were negative for keratin 7, α-fetoprotein, monoclonal carcinoembryonic antigen (CEA), and p53, with a Ki67 index < 5%. The cytoplasm of the hepatocytes was not modified. No keratin 7 positive-billiary channels either portal spaces were present in the tumor parenchyma; the reticulin framework examined with reticulin stain was intact (Figure 2). The surrounding hepatic parenchyma showed a pseudolobular architecture; the pseudonodules of hepatocytes were surrounded by thin fibrous septa that incorporated pseudobiliary channels.

Based on the above findings, the initial diagnosis was HA with clear cell features and peliosis. Based on the patient's age (female >50 years old) and occurrence of the tumor in the setting of cirrhosis (Table 1), the final diagnosis was AHN with peliosis. The HCC was excluded based on identification of one-to-two closely packed rows of proliferated hepatocytes, normal nuclear-to-cytoplasmic ratio, low Ki67 index, and intact reticulin framework. The gallbladder examination confirmed the chronic cholecystitis. The patient was discharged after 1 week of hospitalization and is free of any complaints 6 months after surgery, without any additional therapy.

### Case 2

A 66-year-old nonalcoholic male was admitted at the hospital with severe diffuse abdominal pain. His past medical history included primary hypertension and chronic B hepatitis, without Delta virus. Hepatitis was diagnosed 20 years before and was not treated with any drugs, except hepatoprotective pills. Two years ago, hepatitis-related Laennec atrophic cirrhosis was diagnosed. At the current admission, with no therapy, there were no signs of decompensated cirrhosis.

Physical examination did not reveal significant disorders, except a slight abdominal wall contraction. Ultrasound examination showed a 35 mm solid hepatic nodule in the sixth liver segment. Blood tests did not show significant disorders except slight anemia (hemoglobin 12.00 g/dL and hematocrit 35.20%), and lymphocytosis (46.00%). Transaminases were within normal limits (AST 25.00 U/L, ALT 22.00 U/L) as well as total bilirubin (0.40 mg/dL).

Based on these examinations, laparotomy was decided, and the sixth segment of the liver that embedded a well-defined encapsulated tumor was removed.

Gross examination of the surgical specimen revealed a 35 mm × 30 mm × 38 mm round encapsulated tumor, surrounded by hepatic parenchyma. On cut section, the tumor had a multinodular solid aspect, was green-yellow in color, soft in consistency, and presented multiple small hemorrhagic areas (Figure 1). The liver parenchyma adjacent to the connective capsule had a hard consistency and nodular aspect.

Under microscope, the encapsulated tumor was shown to be composed by multiple nodules separated by connective bands. Within the nodules, hepatocyte-like cells that displayed tubular or glandular-like, acinar and solid-trabecular arrangement of the tumor cells with clear component was noted. No atypical nuclei were identified; the reticulin framework was disintegrated (Figure 2). The tumor stroma was well vascularized, without ductular reaction or inflammatory cells. The hepatic parenchyma that was located adjacent to the connective capsule presented a pseudolobular architecture that was suggestive for cirrhosis, without signs of fatty change. The immunoprofile of the tumor cells was similar to Case 1.

Based on the microscopic aspect, the initial diagnosis was HA with mixed components, including trabecular and acinar structures. Based on the patient's gender (elderly male), the multinodular aspect, 3 hepatocyte-like cells thick plates, many pseudoacini, disintegrated reticulin framework (Figure 2) and associated cirrhosis, the final diagnosis was well-differentiated HCC with peliosis (Table 1). The patient was discharged 9 days after surgery; there were no complaints 7 months after surgery.

## DISCUSSION

HAs and adenoma-like tumors such as AHNs are incidentally discovered during computed-tomography, ultrasound, or magnetic resonance imaging (MRI) examinations. In HAs larger than 70 mm in diameter, spontaneous rupture, intraperitoneal bleeding, and even malignant transformation in HCC can occur,^9^ especially in males.^10^ Despite their apparently benign morphology, AHNs can recur and metastasize and it is likely that malignization of HA is rather an incorrect diagnosed AHNs.^11^ To prevent bleeding and/or consequences of malignant behavior, surgical excision of adenomatous lesions larger than 5 cm is recommended in females, and in males, removal of any tumor nodules, respectively.^6^

In our first case, the risk factors for rupture and/or bleeding in a cirrhotic liver was the diameter that was higher than 70 mm,^12^ but the large peliotic areas can also indicate a high risk for bleeding and during ultrasound examination the tumor can be misconstrued as hemangiomas, such as in this case. Microscopically, in contrast to hemangiomas, the peliotic areas do not display endothelial-lined walls. Although HAs of the left hepatic lobe are more predisposed to rupture,^12^ in this case, the spontaneous bleeding occurred in a tumor that involved the fifth, sixth, and seventh segments of the right-inferior part of the liver.

Based on genetic and pathologic features, 4 major subtypes of HAs are known, with specific histologic features and clinical behavior: inflammatory type (type 1 ∼40–55% of the cases), hepatocyte nuclear factor (HNF-1α)-inactivated (type 2 ∼15–50%) HA, β-catenin-mutated HA (type 3 ∼10–18%), and unclassified HA (type 4 ∼10–30%).^6,10,13–15^ Type 1 HA occurs in patients with a median age of 45 years and is characterized by: 4 to 5 cm nodules composed by proliferation of hepatocyte-like cells, a rich inflammatory stroma with ductular reaction, marked sinusoidal dilatation, and thick-walled arteries. In males with alcoholic cirrhosis, a serum amyloid-A positive inflammatory HA was recently described.^10^ Type 2 HA is usually asymptomatic and occurs almost exclusively in women below 45 years, about 90% of them having a history of contraceptives use. Microscopically, the nodules with a median diameter of 4 cm are composed by hepatocyte-like cells with excessive lipid accumulation in their cytoplasm and can occur on the background of a steatotic liver.^10^ The third type occurs more frequently in young males, is usually associated with male hormones consumption, and the diameter is larger than 15 cm. Microscopically, it is characterized by proliferation of hepatocyte-like cells that can present nuclear atypia and pseudoacinar structures.^6,10,13–15^ Type 4 HA is more frequent in females that can present a history of contraceptives use, the diameter is around 4 cm, can be unique or multiple, and occurs in patients younger than 45 years.^10^

The clinicopathologic characteristics of the first case presented in this article suggested a type 2 HA, an HNF 1-α-mutated subtype,^10^ but its occurrence in an 83-year-old female and development in the background of hepatitis B-related liver cirrhosis indicated an AHN,^11^ based on the criteria synthesized in Table 1. In the second case, which occurred in a 66-year-old male, the pseudoacinar structures suggested a type 3 HA, respectively a β-catenin-mutated HA.^2,10,11^ However, its occurrence in a male with liver cirrhosis and disintegrated reticulin framework asserted for HCC.^11^

**FIGURE 3:**
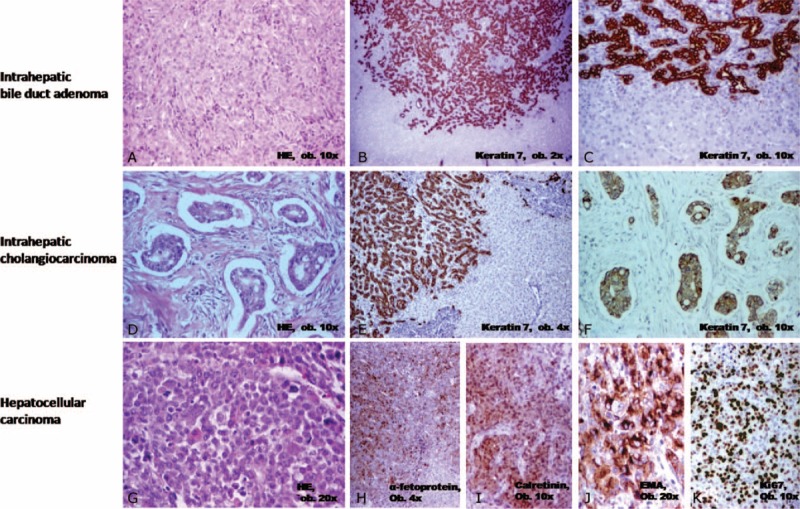
Microscopic and immunohistochemical features of primary hepatic tumors that should be differentiated from hepatic adenomas and adenoma-like tumors.

**FIGURE 4:**
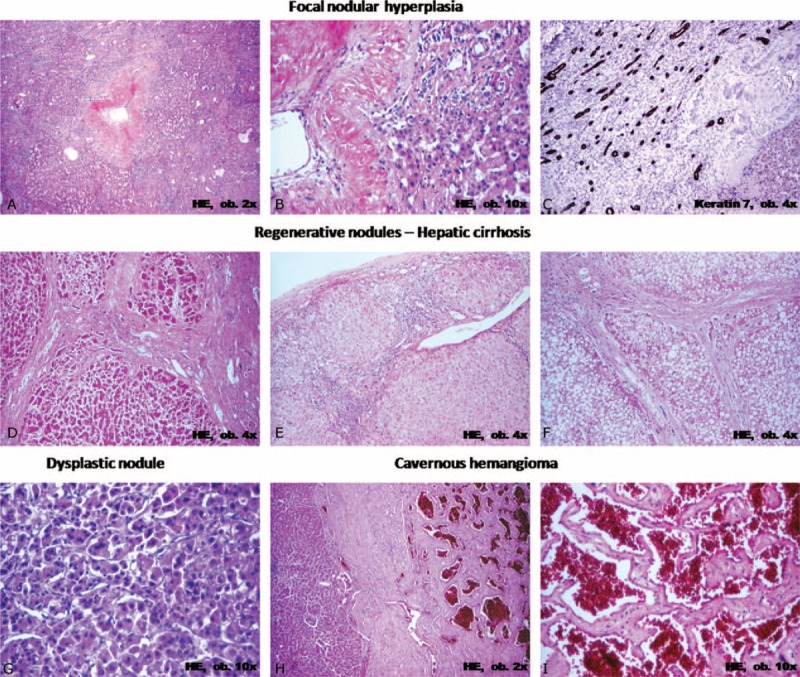
Microscopic and immunohistochemical features of nontumor lesions of the liver that should be differentiated from hepatic adenomas and adenoma-like tumors.

Besides HA, AHNs, and HCC, differential diagnosis of adenoma-like hepatic tumors that occurs in liver cirrhosis should also taken into account other primary hepatic lesions such as focal nodular hyperplasia, dysplastic and regenerative nodules, intrahepatic bile duct adenoma, and cholangiocarcinoma,^16^ whereas peliosis should be differentiated from an associated-cavernous hemangioma (Table 1 and Figures 1–4).

Regarding the preoperative diagnosis, ultrasonography, computed tomography, and MRI examinations are commonly used but differential diagnosis of HA versus HCC is very difficult. Biopsy is necessary for diagnostic confidence and adenomas larger than 4 to 5 cm, in male patients and/or patients with cirrhosis, should be surgically resected.^14^ Moreover, no specific features about differentiation of HA from AHN are known. The MRI is usually used to subclassify HAs in steatotic, peliotic, and mixed (steatotic and peliotic) type.^17^ However, both steatotic and peliotic adenomas shows, according to Lewin et al^17^, hyperintense signal on T1- and T2-weighted images and moderate enhancement at the arterial phase. The steatotic adenomas are usually smaller than 7 cm in diameter, compared to the peliotic type than can be larger.^17^ At contrasting MRI, no delayed enhancement is observed in steatotic type, whereas the peliotic-type adenomas can display persistent enhancement at the portal and delayed phase.^17^ In the present study, both cases were peliotic lesions. In first case, due to large peliotic areas, the ultrasonography revealed the suspicion of hemangioma that was removed due to acute hemoperitoneum. The second case showed, at ultrasonography, a nodule larger than 3.5 cm occurring in the setting of cirrhosis; this was the reason why it was surgically removed without any supplementary investigations.

This article showed 2 unusual cases that highlights the difficulties of diagnosis of tumors with peliosis developed in the setting of cirrhosis. The complex differential diagnosis should be based on the clinicopathologic background, histological aspect, reticulin stain, immunoprofile, and cytogenetic analysis of chromosomal abnormalities. The AHN is a histological entity that should be included and codified in the World Health Organization Classification system.
